# A framework for simulation of temporal evolution and longitudinal studies of breast anatomy in radiology

**DOI:** 10.1002/mp.70207

**Published:** 2025-12-13

**Authors:** Hanna Tomic, Jakob Olinder, John‐Henry Markbo, Pontus Timberg, Sophia Zackrisson, Anders Tingberg, Magnus Dustler, Predrag R. Bakic

**Affiliations:** ^1^ Diagnostic Radiology, Department of Translational Medicine, Faculty of Medicine Lund University Malmö Sweden; ^2^ Department of Imaging and Physiology Skåne University Hospital Malmö Sweden; ^3^ Medical Radiation Physics, Department of Translational Medicine, Faculty of Medicine Lund University Malmö Sweden; ^4^ Radiation Physics, Department of Hematology, Oncology and Radiation Physics Skåne University Hospital Malmö Sweden; ^5^ Medical Radiation Physics, Faculty of Science Lund University Lund Sweden; ^6^ Department of Radiology University of Pennsylvania Philadelphia Pennsylvania USA

**Keywords:** breast imaging, virtual clinical trial, simulation

## Abstract

**Background:**

Virtual imaging trials (VITs) are in silico studies that simulate medical imaging and disease processes, offering a cost‐effective and reproducible addition to traditional imaging trials. While VITs are well established in breast imaging, most existing implementations simulate imaging based on static anatomical models, capturing only a single time point. This limits their ability to study time‐dependent processes such as tumor progression or breast density and composition changes over time.

**Purpose:**

We introduce STELLA‐R (***S*** *imulation of* ***T*** *emporal* ***E*** *volution and* ***L*** *ongitudina* ***L*** *studies of breast* ***A*** *natomy in* ***R*** *adiology)*, the first framework aimed at performing longitudinal virtual imaging trials in breast imaging. STELLA‐R is designed to simulate temporal changes in breast anatomy, density, and lesion development across a virtual population of women.

**Methods:**

Our simulation pipeline consists of five modules. The population creator module generates realistic virtual cohorts based on real‐world data from approximately 25 000 women, modeling multivariate distributions of age, breast shape, and breast density. The phantom creator and lesion creator modules enable detailed specification of breast and lesion characteristics, utilizing Perlin noise computational algorithms to replicate tissue appearance. The tumor location is assigned in the lesion insertion module. To simulate temporal changes in the breast, we used real‐world data from two consecutive screening rounds. This enabled realistic modeling of mammographic density evolution, breast volume changes, and tumor growth. Different breast densities were achieved by adjusting threshold values applied to the Perlin noise, which determines the amount of tissue structure. Temporal changes of parenchyma were simulated by gradually varying the threshold values. Tumor progression was simulated by increasing lesion size according to growth rates sampled from real‐world data. Lastly, the Image Generation module integrates multiple external software components for mammographic image formation, including noise and scatter simulation and image reconstruction. In this study, we simulated digital breast tomosynthesis (DBT) images of our phantoms using open‐source tools. Our simulation framework is modular and can be extended to support additional imaging modalities.

**Results:**

We demonstrate case examples of virtual women at ages 40, 57, and 74, reflecting Swedish screening intervals, and report simulated changes in volumetric breast density over time (14%, 9%, and 6%, respectively). The breast density is modeled with a mean accuracy of < 2% compared to target values. Additionally, we illustrate lesion progression across multiple time points, assuming a tumor doubling time of 282 days. Our fitted models accurately capture correlations between age, breast volume, density, and annual changes.

**Conclusion:**

STELLA‐R pipeline provides a novel foundation for evaluating long‐term screening strategies, imaging, and risk models in a controlled and customizable manner using longitudinal VITs.

## 1 INTRODUCTION

Population‐based breast cancer screening programs are designed to detect cancers at an early stage and thereby reduce breast cancer mortality. Digital mammography (DM) remains the gold standard for screening, although digital breast tomosynthesis (DBT) is being introduced as an alternative. While these imaging modalities are central to screening programs, their effectiveness can vary. The sensitivity of mammography, for example, is influenced by several factors. One key factor is mammographic breast density, the proportion of dense fibroglandular tissue relative to fatty tissue visible on a mammogram. Breast density changes with age and directly affects how well tumors can be detected. Evaluating the impact of breast density and tumor visibility on screening sensitivity in clinical trials is challenging, as traditional study designs are often limited by cost, time, and ethical considerations. These considerations make it difficult to assess long‐term screening outcomes, evaluate new imaging technologies, and compare screening strategies across diverse populations.

Virtual imaging trials (VITs), also referred to as virtual clinical trials or in‐silico trials of medical imaging, have emerged as a powerful alternative. VITs enable researchers to simulate different populations (screening or patients) and imaging scenarios in a controlled and reproducible way.^1^, ^2^, ^3^ In particular, longitudinal VITs, which follow virtual individuals over time, have the potential to revolutionize how we evaluate imaging performance, risk models, and intervention outcomes. By simulating the anatomical and physiological changes that occur as women age, these trials provide valuable insights into breast tissue evolution. For example, the breast anatomy can change due to hormones, menopause or simply by the process of aging that leads to tissue involution, where dense fibroglandular tissue is gradually replaced by fatty tissue. This transformation influences cancer detectability and risk assessment.

Despite recent progress in generating realistic breast phantoms, most existing VITs in breast imaging rely on static snapshots or simulate only a single time point. These approaches fail to capture the temporal dynamics of breast tissue involution, parenchymal complexity, and tumor development. Simulating these processes over time remains a significant challenge, in part due to the lack of accessible longitudinal mammographic datasets and the complexity of accurately modeling tissue variability.

To address this gap, we build on our earlier work using Perlin noise algorithms,^4^, ^5^, ^6^ originally developed for computer graphics,^7^, ^8^ to simulate realistic breast parenchyma and lesions. This technique enables the creation of 3D voxel‐based breast phantoms that can be adapted to reflect age‐dependent changes in anatomy and linked to population‐level data on density and tumor characteristics. Furthermore, these phantoms are compatible with multiple imaging modalities (e.g., computed tomography, DM or DBT), allowing their use in diverse simulation environments.

This study aims to develop and demonstrate a simulation framework, STELLA‐R, for longitudinal virtual imaging trials in breast imaging, capable of modeling temporal changes in breast anatomy and enabling the investigation of how these changes impact tumor visibility, breast density, and imaging performance over time.

## 2 MATERIALS AND METHODS

### 2.1 The framework of the STELLA‐R simulation pipeline

**FIGURE 1:**
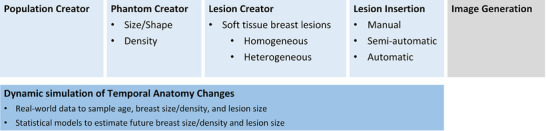
Schematic of the simulation framework: The simulation begins by generating a population, followed by the creation of phantoms and lesions based on the population's characteristics. Lesions can be inserted in a manual, semi‐automatic, or automatic workflow. Temporal changes in the breast tissue and lesions can be simulated as well. Additional open‐source tools have been incorporated with the framework to visualize the phantoms in simulated x‐ray images (image processing), by creating projections, adding system noise, and reconstructing the images.

We are presenting a framework for simulating the natural progression of breast tissue and breast lesions over time. The outline of the simulation framework is presented in Figure 1. Our framework consists of five modules: population creator, phantom creator, lesion creator, lesion insertion and image generation. The first four modules are in‐house developments. The Image Generation module is an integral part of our simulation framework, utilizing open‐source tools to simulate x‐ray projections, incorporate system noise, and perform DBT reconstruction.^9^, ^10^, ^11^ The simulation of temporal changes in the breast tissue and lesions is detailed in Section 2.2.

### 2.2 Population creator

Our simulation framework can generate dynamic phantoms and lesions with characteristics based on data from real‐world populations. In this paper, we describe the simulation of breast volume, breast density and lesion size, evolving over time. We use data from the reference population of the Malmö Breast Tomosynthesis Screening Trial (MBTST), one of the early European studies of DBT clinical performance conducted at our institution.^12^, ^13^ We refer to the reference population as the real‐world DM screening population. In addition, we incorporate data from a separate cohort in Malmö, Sweden, which includes measurements of tumor size and tumor volume doubling time measured at two separate time points.^14^ In previous work, we demonstrated how these data can be applied to generate virtual women with corresponding tumor characteristics.^15^

The population creator module operates by importing tabulated data from real‐world (or virtual) populations to generate representative probability functions. The probability functions then serve as the basis for sampling the virtual cases. Further details on virtual population generation and sampling process are provided in the Section 2.1 “creation of a virtual population”.

Once the virtual population is sampled, the characteristics of each case evolve over time according to statistical models that are derived from the two imported datasets. The datasets describe longitudinal changes in breast volume, breast density, and lesion size. The resulting models enable simulations of breast development and disease progression, as detailed in Section 2.2.

The population creator supports two modes of operation. Users may either (i) generate cases automatically based on population‐derived characteristics from tabulated datasets, or (ii) manually specify breast and lesion parameters such as volume, density, and size. By default, the population creator includes the real‐world DM screening population and the Malmö tumor cohort, but the module can be modified to incorporate other datasets.

### 2.3 Real‐world DM screening population

We have analyzed data on breast volume, dense volume, volumetric breast density percentage (VBD%), and age at two consecutive screening rounds from the real‐world DM screening population. This population includes all women that participated in the Malmö breast screening program between 2010 and 2015, with two view DM. The breast screening program in Sweden recommends intervals of 18 months for women aged 40–54 and 24 months for those aged 55–74. We included women with a maximum interval of 30 months between two successive screening examinations, to reduce likelihood of including women who missed a screening round. For women with more than two screening rounds during the study period, only the first two were considered. This ensures consistency across all participants, improving the stability and comparability of the model.

Most images (91%) were acquired using a Siemens Mammomat Inspiration system (Siemens Healthineers, Erlangen, Germany), while the remainder were obtained with either the Siemens Mammomat Fusion or Mammomat Novation DR systems. Only for‐presentation images were available.

After excluding women with breast implants and those with invalid or missing density data, the final sample consisted of 25–188 women. Breast volume and VBD% were assessed separately for the left and right breast using a commercial AI software (Transpara Version 2.0.0, ScreenPoint Medical, Nijmegen, Netherlands)^16^, ^17^ on the DM images. For each examination, the AI software selects the highest breast volume and highest VBD% from the two breasts to represent the case. This selection is in accordance with ACR BI‐RADS, the standard reporting system, which states that the denser breast should determine the breast density category.^18^ Absolute dense volume was calculated by multiplying the total breast volume by the corresponding VBD%. This value represents the estimated volume of fibroglandular (dense) tissue within the breast, which is an important factor influencing lesion visibility and radiation dose modeling in breast imaging simulations.

### 2.4 Creation of a virtual population

We fitted a multivariate distribution, a t‐copula, to model the dependence between five variables: the breast volume, dense volume, age, change in breast volume, and change in dense volume. Changes in breast volume and breast density were normalized relative to the change in age between the two imaging occasions for each woman. A copula is a mathematical function that combines individual distributions into a unified multivariate distribution.^19^ Copulas are useful for modeling dependencies between variables with non‐normal distributions, such as the breast volume and dense volume from the real‐world DM screening population. The t‐copula is particularly robust, as it effectively captures tail dependencies and extreme co‐movements. We used MATLAB's (MathWorks Inc., Natick, MA, USA) built‐in *copulafit()* function for the analysis. Using this approach, the virtual population was generated from the fitted distributions rather than copying the original dataset directly, ensuring that the virtual population reflects the key statistical properties of the real‐world data. This method introduces natural variability, allows the generation of larger populations, and enables exploration of variations while preserving the overall characteristics of the source data.

To prevent negative values in the samples generated from the fit, breast volume and dense volume were log‐transformed before fitting and back‐transformed afterward. Additionally, a kernel smoothing function was applied to produce smoothed cumulative distribution functions (CDFs), which serve as essential inputs for MATLAB's built‐in *copulafit()* function. The fitted t‐copula is described by the correlation between the variables, in a correlation matrix $\hat{\rho }$, and by a degree of freedom $\hat{\nu }$ that models tail dependence. These values can be used as input to reproduce the same t‐copula distribution. The parameters were estimated via maximum likelihood estimation.

We fitted a copula model for the entire population, as well as separate copulas for each individual age between 40 and 74. The methodology was consistent across all models, with the difference being that the data were stratified by age. To account for age variability, we included a tolerance window of ± 0.5 years around each target age (e.g., 39.5–40.5 for age 40). This tolerance window was selected due to that we observed some women being invited for screening slightly before turning 40 years. The individual copulas allow for realistic simulation of breast volume, dense volume, and their respective changes tailored to each specific age group. It is particularly useful when users wish to generate virtual data for customized populations spanning different ages. Users can specify the target age and the desired number of virtual samples (i.e., virtual individuals) for simulation.

Similarly, as described in our previous work,^15^ we fitted models to data on tumor size and tumor volume doubling time—which are then used to sample these tumor characteristics for each virtual patient with a tumor.

### 2.5 Comparison of real‐world and virtual populations

Goodness‐of‐fit was assessed by visually and quantitatively comparing real‐world versus simulated data distributions. Histograms were used to compare the distributions of individual variables, and scatterplots were used to compare correlations between real‐world versus simulated values.

We sampled 100 simulated datasets (virtual populations) of the same size as the real‐world data. Marginal distributions were evaluated by comparing the mean and standard deviation (SD) of each variable across simulated replicates versus the corresponding real‐world means and SDs.

To assess the multivariate dependence structure, we computed the pairwise Kendall's tau correlations for all variable pairs in each simulated dataset (*τ*_s_). Across the 100 replicates, we calculated the mean and 95% confidence interval of the simulated *τ*_s_ values for each pair and compared these versus the real‐world *τ*_r_ values to evaluate how accurately the copula captures the dependence structure.

### 2.6 Phantom creator

Users can select the shape, volume, and volumetric breast density of the breast phantom, and adjust the appearance and distribution of glandular and adipose tissue.

**FIGURE 2:**
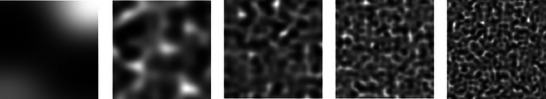
From left to right are examples of different Perlin noise frequencies, that are later added together (superimposed) to create a realization of layered breast tissue. Two crucial parameters are used to alter the noise; lacunarity—which determines the increase in frequency, and persistence—which controls the amplitude.

We simulated voxel‐based phantoms based on a modified in‐house implementation of the Perlin noise algorithm.^4^, ^5^ Simulated continuous tissue structures were defined by applying random noise gradients in a grid structure. We superimposed several octaves of different spatial frequencies (Figure 2) to generate a realization of layered breast tissue.

In Perlin noise, lacunarity and persistence control the structure of the output: lacunarity controls the change in frequency between each octave, while persistence determines the change in amplitude. Together they control the size range and variation in structures. These parameters were fine‐tuned to match breast tissue, using results from previous studies.^4^ A tunable threshold is applied to the simulated structures, controlling the thickness of the generated elongated structures. The threshold value can range between zero and one. A value of one corresponds to zero thickness, that is, no structures are created. A value of zero creates very large structures that cover the entire simulated volume. The threshold value controls the basic tissue appearance and density, but because of the random nature of Perlin noise, any number of unique simulated breasts can be generated by reusing the same threshold values with different randomly selected seeds.

Tissue structures were simulated in 2200 × 1200 × 600 voxel blocks with a default 0.1 mm resolution, adjustable by the user. The block size was chosen to fit the largest breast outlines,^20^, ^21^ allowing consistent masking of the breast volume and standardization of the simulation framework. Several 3D blocks of simulated tissue, with different individual parameters, make up each complete breast tissue model. Each voxel was assigned a specific tissue index, representing a set mix of glandular and adipose tissue. Each voxel was then mapped to corresponding attenuation values in OpenVCT, an open software framework for virtual imaging trials.^9^

In this paper, we evaluated how thresholding the Perlin noise influences simulated breast density. Specifically, we systematically varied the threshold values by scaling them with a scaling factor (SF). We selected five representative SF values for evaluation, thus generating five unique threshold levels. For each new threshold level, we generated ten independent breast phantoms and calculated the percent dense tissue. To assess variation, we report the mean and standard deviation of breast density across the ten phantoms at each threshold level. This approach allowed us to characterize both the overall effect of thresholding on density and the consistency of simulated density within each threshold level.

The breast density in the simulated breast phantoms is defined by (1) summing up individual voxels, multiplied by their material weight (percent adipose and glandular tissue), and (2) dividing the obtained sum of glandular volume by the total volume (sum of all voxels) to get the percentage of glandular tissue.

### 2.7 Lesion creator

Simulated breast lesions were generated using our modified Perlin noise algorithm. In our previous work, we developed several lesion simulation methods ranging from homogeneous to heterogeneous compositions.^22^ One method produces irregular soft‐tissue breast lesions with homogeneous, uniform material properties, while others generate more heterogeneous structures by enhancing existing background tissue and incorporating additional Perlin noise frequencies into the lesion model.

By altering the simulation parameters, we can represent a variety of soft tissue breast lesions with different characteristics, such as size, shape, and the appearance of lesion edges. The size of the lesions and their growth rate (in terms of tumor volume doubling time) can be determined in the population creator module, as these characteristics are sampled from real‐world data. The tumor shape and edges are defined by the Perlin noise algorithm.^6^, ^22^ Tumor shape and edge appearance can only be specified manually and are not based on specific characteristics from a real‐world population. Consequently, only lesion size and growth rate are forwarded from the population creator module to the lesion creator module, although users can also manually specify these parameters if desired. The population creator is designed to centralize the handling of real‐world datasets, allowing users to easily change the source data without introducing inconsistencies between population characteristics and simulated lesions.

### 2.8 Lesion insertion

There are three modes of lesion insertion within the framework: (i) manual, (ii) semi‐automatic, and (iii) automatic.

- **Manual insertion**
  A slice in the *z*‐direction (transverse plane) of the voxelized phantom is first selected to define the plane intersecting the center of the lesion. This slice is then displayed to the user for visualization. From the displayed image, the user interactively selects the *x*‐ and *y*‐coordinates of the lesion center.
- **Semi‐automatic**
  The user selects the center coordinates (*x*, *y*, *z*) of the lesion within the breast volume. One or more of these coordinates can be fixed manually, while the remaining coordinates are randomly assigned within predefined ranges. For example, choosing the “fixed depth” option keeps the *z*‐coordinate constant, whereas the “varied depth” option allows the *z*‐coordinate to vary randomly within a specified range. The semi‐automatic mode is available when the user wants to avoid the interactive prompt that is presented in the manual insertion mode. The semi‐automatic mode, therefore, serves as a faster alternative for large‐scale lesion generation.
- **Automatic**
  For the automatic mode, we developed a method to realistically position the lesion within the volume, based upon clinically observed lesion locations,^19^, ^20^, ^21^ with minimal user intervention. The breast volume was divided into four quadrants by two reference lines. First, a horizontal line that separates the upper and lower halves. Second, a vertical line that separates inner (medial) and outer (lateral) halves, intersecting at the nipple. The quadrants were referred to as upper outer (UO), upper inner (UI), lower outer (LO), and lower inner (LI), according to clinical standards. Each quadrant was assigned a probability of having a lesion, set to 58.5% (UO), 15.7% (UI), 8.4% (LO), and 9.9% (LI). The probabilities are based on the mean value from three clinical observations.^23^, ^24^, ^25^ Each voxel value in the breast phantom is mapped against a weighted mixture of adipose and glandular tissue. Once the quadrant is determined, the precise insertion coordinates within the phantom are automatically selected based on the assumption of a linear relationship between lesion position and breast density, that is, the proportion of glandular tissue in the phantom. Lastly, we restricted the lesion insertion to be within a bounding box, defined as a mask covering 90% of the phantom volume. This ensures that lesions are not placed subcutaneously, as such positioning is unlikely in real clinical scenarios.

### 2.9 Image generation and technical specifications

To simulate variability in breast positioning during imaging, the 3D volumes can be automatically rotated around the *x*‐, *y*‐, and *z*‐axes by randomly selecting small rotation angles, adjustable by the user. Rotations are performed using nearest‐neighbor interpolation to preserve the original voxel values. We simulated examples to illustrate how repositioning relative to the imaging system can change the appearance of the same breast volume. To clearly highlight tissue structure changes over time, we also simulated breast volumes that remain fixed across imaging sessions.

We have used the open‐source software, OpenVCT,^9^ to simulate x‐ray projections of the voxel‐based phantoms. System noise (Poisson and electronic) was added to the projections using methods developed by Borges et al.^11^ DBT projections were reconstructed using filtered back projection, following the method developed by Vimieiro et al.^10^ We used the noise profile and geometry of a Siemens mammography unit with a reconstructed pixel size of 0.085 mm and slice thickness of 1 mm. Radiation scatter from the primary x‐ray beam can also be incorporated using scatter factors produced for both DM and DBT systems by Diaz et al.^26^ To demonstrate our pipeline for simulating an aging population, we chose not to include scatter effects, as they would not significantly contribute to this demonstration. If the user wants to add scatter, we recommend applying the scatter kernel immediately after projection and before adding system noise. This is due to that both scatter and primary photons are generated prior to noise in the imaging chain. This preserves the physical order of events in the simulation. In addition, the MeVIC software (Barco Healthcare) ,^27^ integrated into the OpenVCT framework, can be used to simulate human image interpretation using a Channelized Hotelling Observer (CHO) model.^28^ It incorporates detailed display characteristics, including grayscale calibration, luminance response, modulation transfer function, noise, and angular dependency.^29^, ^30^ MeVIC supports repeated CHO evaluations, enabling estimation of diagnostic performance through ROC analysis.^31^

All simulations were performed in MATLAB version 2024b (Mathworks, Natick, Massachusetts, US) on a workstation equipped with an AMD Ryzen Threadripper 2970WX 24‐Core Processor (Advanced Micro Devices, Santa Clara, California, US), 256 GB RAM, and four NVIDIA GeForce RTX 2080 Ti GPUs (NVIDIA Corporation, Santa Clara, California, US).

## 3 SAMPLE APPLICATIONS

### 3.1 Breast density changes over time

To illustrate possible applications of the proposed framework, we simulated breast density changes in an aging population of women. Several studies have examined age‐related changes in breast tissue. These studies show that mammographic breast density declines over time due to tissue involution, and that the rate of change is influenced by menstrual and reproductive history, menopausal status, and hormonal exposures.^32^, ^33^, ^34^, ^35^ Recently, our lab has investigated the temporal changes in volumetric breast density in one screening round.^36^ The density data were obtained from the real‐world DM screening population, detailed in Section 2.1.^12^ We have developed three methods for simulating temporal changes in breast density. Comparing the methods requires additional longitudinal follow‐up data from the screening cohort, which is currently being collected. Consequently, the results are focused primarily on Method A, while Methods B and C are still under refinement and proposed as future directions.

**FIGURE 3:**
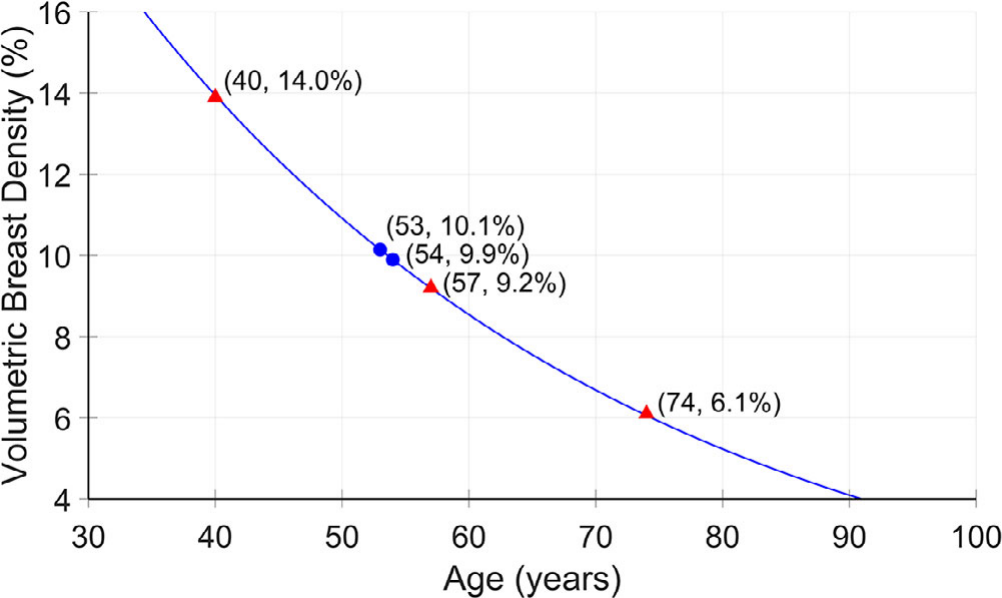
Assuming an exponential relationship between a woman's age and volumetric breast density (VBD%), the red markers indicate VBD% values at key screening milestones: age of inclusion, midpoint, and exclusion from the screening program (40, 57, and 74 years, respectively, in Sweden). The blue markers represent the mean age and mean VBD% of the population (53 years, 10.14%) and the corresponding values after applying the average annual change in VBD% (54 years, 9.89%). These points were used as baseline inputs for fitting the exponential model.

- The first approach, Method A, involves adapting a curve based on data described in a study by Olinder et al. to model breast density changes over time.^36^ While the original data focused on the same population, slight differences exist in the included participants due to differences in selection criteria, described in Section 2.1. We computed the average annual change in VBD% for the entire population, which was approximately −0.25 percentage points, with a mean age of 53 years and a mean VBD% of 10.14%. Using this annual change rate (illustrated by the blue markers in Figure 3) and by assuming an exponential relationship between the woman's age and VBD%, we could model the breast density at key screening ages: baseline (40 years), midpoint (57 years), and end of screening (74 years), as shown by the red markers in Figure 3.
- It is known that density does not decline at a constant rate across all ages.^37^ To account for this, in the second approach we stratified the population by age and estimated the average change in VBD%, expressed in percentage points, within each age group, as shown in Table 1. Changes in breast volume and breast density were normalized relative to the change in age between the two imaging occasions for each woman. In the longitudinal simulation of the virtual population, the change VBD% is updated as the simulated subject transitions from one age group to the next. Each age group is associated with a separate exponential model, so changes in VBD% over time are applied in a stepwise manner based on the corresponding model for that age range. This approach results in piecewise exponential changes in VBD%, rather than fitting a single curve across the entire age span (as done for Method A).
- The third approach for simulating change in breast density on an individual level is to sample the change in breast volume and change in breast density from the t‐copula distribution mentioned in Section 2.1, “creation of a virtual population”. This approach is suitable when simulating two consecutive screening rounds. For longitudinal studies, spanning over several screening rounds, the adapted curves from Methods A and B are more appropriate. They represent an average decline across all women in a specific age group or over all age groups, rather than sampling changes at the individual level. This avoids the accumulation of random variation across multiple screening rounds and better reflects the systematic, population‐level trend observed in the real‐world data. The choice between Method A and Method B depends on the desired level of complexity.

**TABLE 1:** Clinically observed changes in VBD% per year for different age groups.

Age group	Group count	Change in VBD% per year (percentage points)
40–44	6515	−0.27
45–54	8819	−0.38
55–64	6263	−0.15
65–74	3591	−0.028

### 3.2 Breast volume changes over time

**FIGURE 4:**
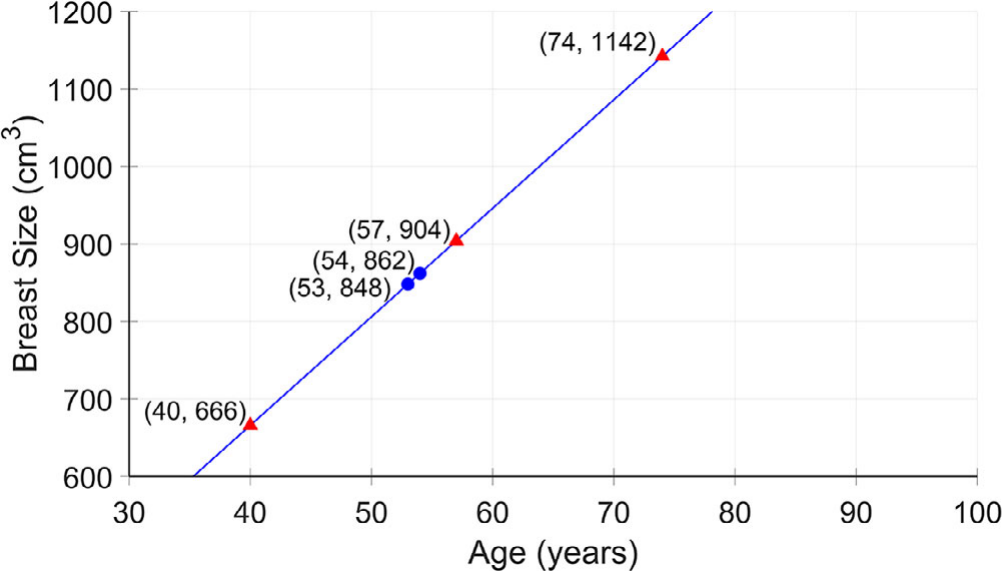
Assuming a linear relationship between the woman's age and the breast volume. Shown as red markers is the breast volume at the age of inclusion, midpoint, and exclusion from the screening program (40, 57, and 74 years, respectively, in Sweden). The linear model was estimated based on the average breast volume of 848 cm^3^ at the mean age of 53 years, with an annual change in volume of 14 cm^3^ observed in the real‐world DM screening population—this is represented by the blue markers.

Similarly to the methodology explained for simulating the change in breast density over time, we can also simulate the change in the total breast volume over time using Methods A‐C. The data were calculated from the real‐world DM screening population, described in Section 2.1.^12^ The mean average volume increase per year across all ages was 14 cm^3^. We assumed a linear relationship between age and breast volume. If the average breast volume corresponds to the average age (53 years, 848 cm^3^), we can estimate breast volume at both inclusion, midpoint, and exclusion from the screening program as seen in Figure 4.

If opting for Method B, the average change in breast volume for women in different age groups is displayed in Table 2. As mentioned earlier, piece‐wise linear or other functions can be fitted to the individual age intervals to model the breast volume changes over time. The breast volume can also be extracted from the t‐copula distribution, as mentioned in Method C.

**TABLE 2:** Clinically observed changes in breast volume per year for different age groups.

Age group	Group count	Change in total volume per year (cm^3^)
40–44	6515	16.64
45–54	8819	18.43
55–64	6263	11.68
65–74	3591	0.60

### 3.3 Breast tumor changes over time

**FIGURE 5:**
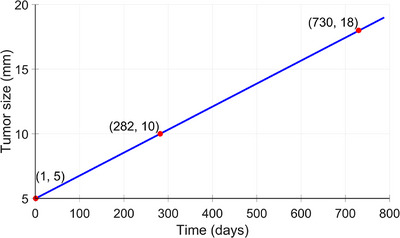
Assuming a linear increase in tumor diameter over time, we simulated the tumor growth at three time points: (i) the initial diameter of 5 mm, (ii) after one tumor volume doubling time (assumed as 282 days), corresponding to the diameter of 10 mm, and (iii) after one screening round, (730 days = 2 years), corresponding to the diameter of 18 mm.

Another sample application is the simulation of growing tumors. Previously, we have utilized real‐world data from MBTST and another cohort from Malmö to build population‐based probability functions of lesion size and tumor growth.^14^, ^15^, ^38^ In this study, as an example, we use the mean tumor volume doubling time (282 days, range 46–749 days) as observed by Förnvik et al.^14^ to simulate the growth of a soft tissue breast lesion, starting from 5 mm in diameter (Figure 5). In this example, we simulated the lesion according to the third lesion simulation method, described in Section 2.1. “lesion creator”, which both enhances the background tissue and includes additional Perlin noise frequencies within the lesion.

## 4 RESULTS

Using the computer system described in Section 2.1“image generation and technical specifications”, it took on average 46 ± 2 min to generate and save a Perlin noise phantom of 2200 × 1200 × 600 elements, irrespective of the breast density. The time to generate the tumor, insert it in the phantom, and save the file varied depending on the tumor size, for example, it took on average 47–82 s to simulate tumors with a diameter ranging between 2–20 mm.

### 4.1 Virtual population

**FIGURE 6:**
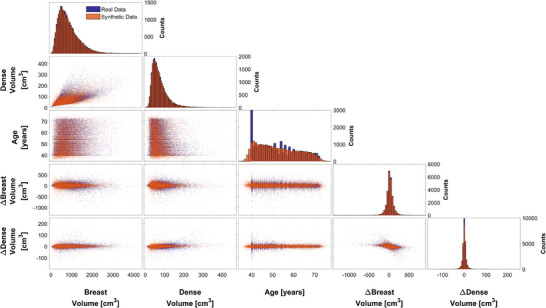
Pairwise matrix plots for the real‐world data (blue) and simulated data (orange). Diagonal panels display histograms of each variable, with bar heights indicating counts. Off‐diagonal panels show scatter plots for each pair of variables. Several variable pairs display clear tail dependencies.

We compared real‐world and virtual populations by scatter plots between all variables (breast volume, dense volume, age, change in breast volume, and change in breast density), as can be seen in Figure 6.

The two data sets shown in Figure 6 can be visually compared to assess whether the real‐world population has been accurately modeled. The input parameters for the t‐copula model are shown in Table 3, that is, the correlation matrix $\hat{\rho }$. The degrees of freedom $\hat{\nu }$ was estimated to 16.44.

**TABLE 3:** Estimated correlation matrix for the five variables (the breast volume, dense volume, age, change in breast volume, and change in breast density) based on the fitted t‐copula model. The matrix describes the dependence between variables. Each entry represents the copula‐based correlation $\rho_{\mathrm{ij}}$ between variable *i* and variable *j*. The diagonal entries are 1, indicating perfect self‐correlation in order from left to right: Breast volume, dense volume, age, change in breast volume, and change in breast density. The off‐diagonal values range between ‐1 and 1, with higher absolute values indicating stronger dependence.

Variable	Breast volume	Dense volume	Age	Change in breast volume	Change in breast density
Breast volume	1				
Dense volume	0.64	1			
Age	0.18	−0.16	1		
Change in breast volume	−0.05	−0.04	−0.07	1	
Change in breast density	0.07	−0.20	0.02	−0.52	1

The quantitative comparison of the marginal distributions between the real‐world and simulated data is shown in Table 4. Table 5 presents the pairwise Kendall's *τ* correlation for all variable pairs. For each pair, the real‐world *τ*_r_, the mean simulated τ_s_ across 100 bootstrap replicates, the 95% confidence interval of the simulated τ_s_ and the mean difference between *τ*_r_ and *τ*_s_ (Δ*τ*) are shown.

**TABLE 4:** Comparison of marginal distributions between the real‐world and simulated data. For each variable, the mean and standard deviation (SD) are reported for the real data and for the simulated datasets (averaged across 100 bootstrap samples).

Variable	Real‐world: Mean + SD	Simulated: Mean + SD
Breast volume [cm^3^]	848 ± 495	851 ± 504
Dense volume [cm^3^]	74 ± 42	74 ± 42
Age [years]	53 ± 10	53 ± 10
ΔBreast volume [cm^3^]	14 ± 95	14 ± 96
ΔBreast density [cm^3^]	0.5 ± 11	0.5 ± 11

**TABLE 5:** Kendall's tau correlations for each variable pair in the real‐world dataset (*τ*_r_) and the mean correlations from 100 bootstrap samples of simulated data (*τ*_s_), including 95% confidence intervals (CI) of *τ*_s_ (CI lower—CI upper). The mean difference, Δ*τ*, between real and simulated correlations is also provided for each pair.

Variable pair	Real‐world τ_r_	Mean simulated τ_s_	95% CI of simulated τ_s_	Mean difference Δτ
Breast volume;dense volume	0.444	0.446	0.438 – 0.452	0.001
Breast volume;Age	0.112	0.116	0.109 – 0.123	0.004
Breast volume;Δbreast volume	−0.024	−0.032	(‐0.040)—(‐0.025)	−0.009
Breast volume;ΔBreast density	0.044	0.047	0.038 – 0.055	0.003
Dense volume;Age	−0.103	−0.101	(‐0.108)—(‐0.092)	0.002
Dense volume;Δbreast volume	−0.012	−0.022	(‐0.030)—(‐0.014)	−0.010
Dense volume;Δbreast density	0.126	0.126	0.118 – 0.134	0.000
Age;Δbreast volume	−0.048	−0.046	(‐0.055)—(‐0.038)	0.001
Age;Δbreast density	0.009	0.012	(0.002)—(0.021)	0.003
ΔBreast volume;Δbreast density	−0.350	−0.348	(‐0.356)—(‐0.340)	0.002

**FIGURE 7:**
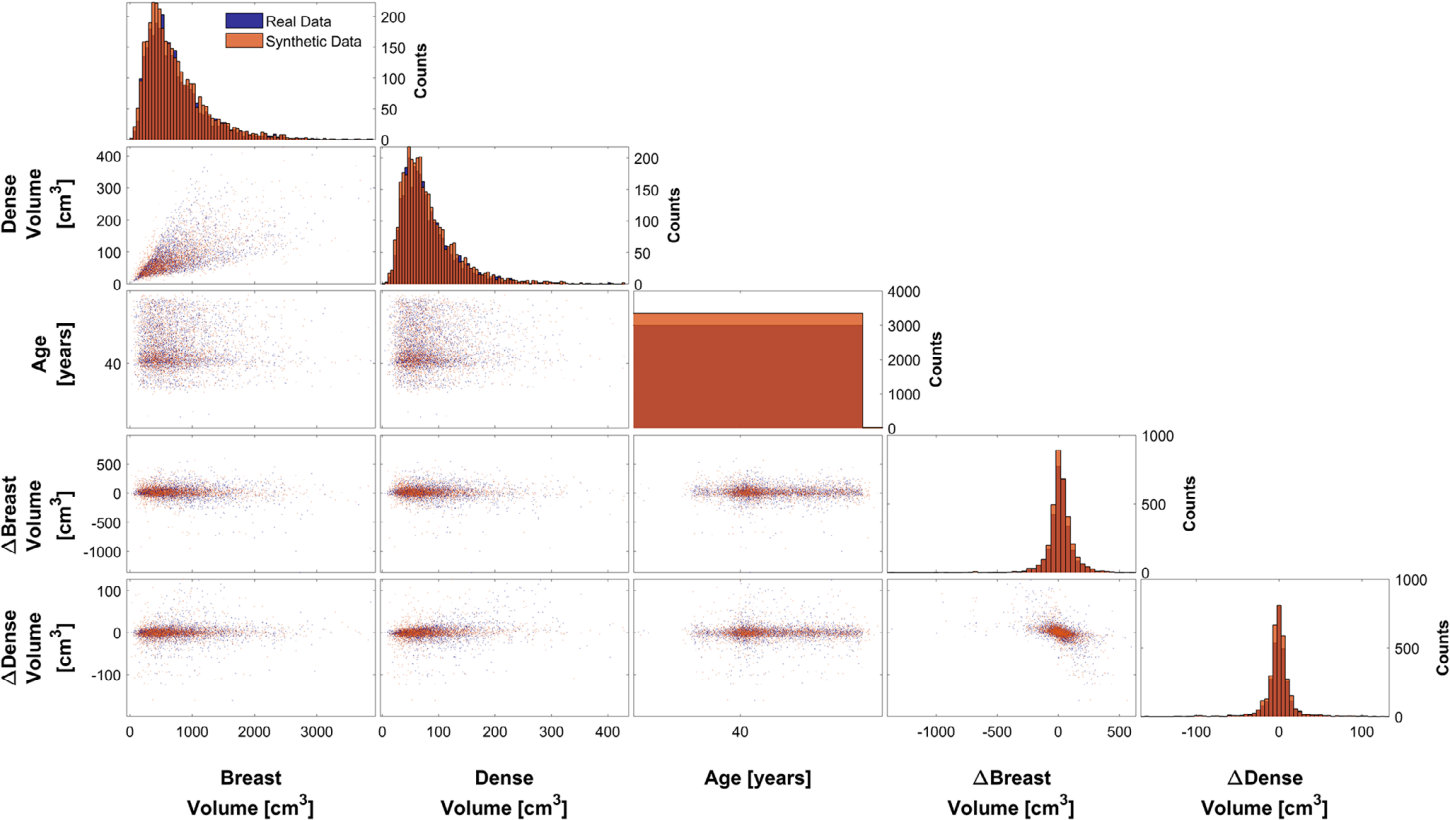
Pairwise matrix plots for the real‐world data (blue) and simulated data (orange) for women aged 40 years. Histograms of the individual variables are shown along the diagonal, where bar heights indicate counts. Scatter plots of each variable pair are presented in the off‐diagonal panels. Clear tail dependencies are visible in several variable pairs.

We also fitted individual copulas for each age between 40 and 74 years. As an illustrative example, Figure 7 presents the results for the real‐world and virtual populations for women aged 40 ± 0.5 years. While the same methodology was applied across all age groups, only the results for age 40 are shown here for simplicity.

### 4.2 Phantom creator

**FIGURE 8:**
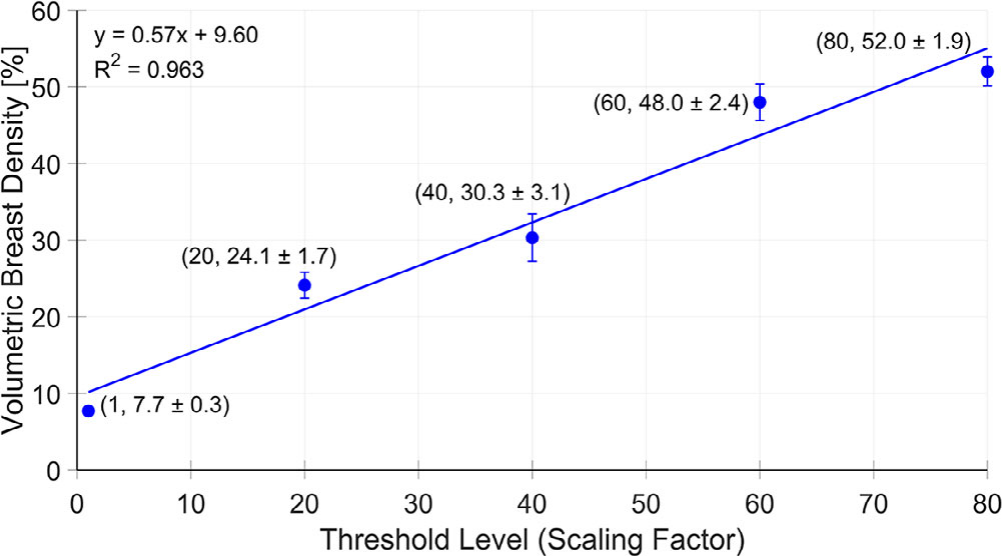
The average volumetric breast density and standard deviation for five different Perlin noise threshold levels. A curve has been fitted to enable interpolation of threshold levels/scaling factors for given densities.

Figure 8 shows the mean and standard deviation of VBD% across the five Perlin noise threshold levels, calculated from the simulated phantoms. The average deviation in VBD% across all threshold levels was 1.9%.

### 4.3 Lesion insertion

**FIGURE 9:**
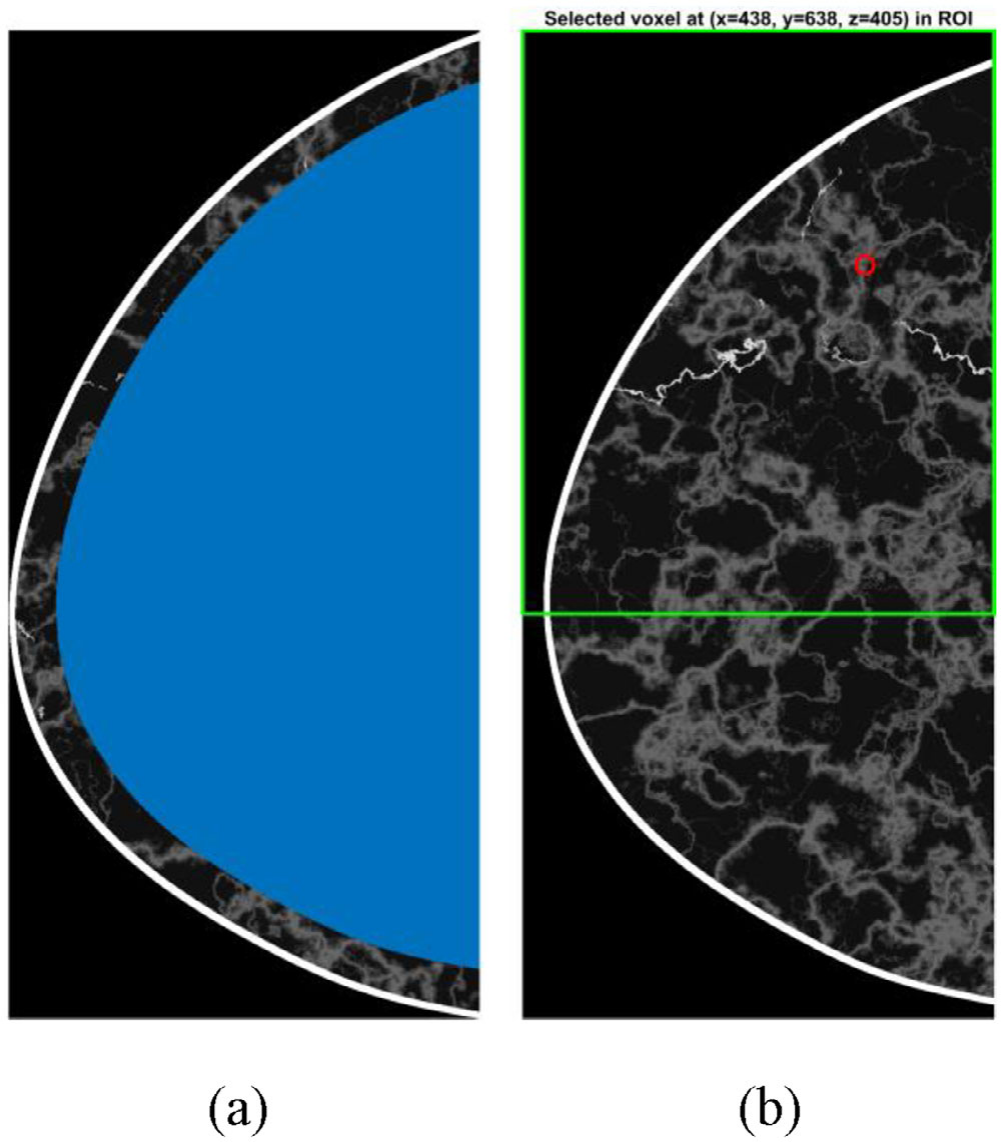
Visualization of the mask used to prevent insertion of subcutaneous lesions in (a). The blue area indicates the permitted insertion region. In (b), an example insertion location is shown in red, along with the corresponding selected quadrant highlighted in green.

We demonstrate an example of the insertion of lesions in Figure 9a,b.

### 4.4 Breast density changes over time

**FIGURE 10:**
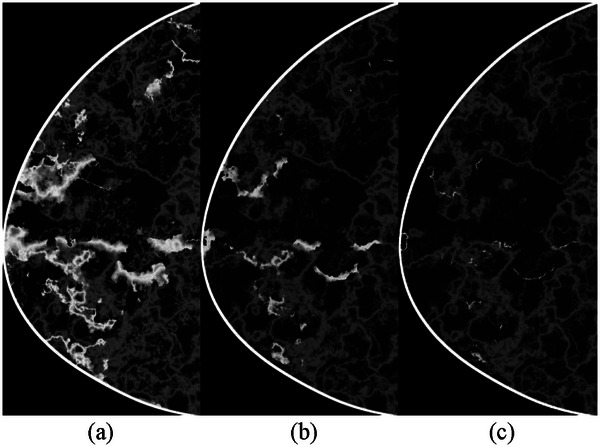
Cross‐section of the voxel‐based breast phantom of a virtual woman at (a) 40, (b) 57, and (c) 74 years of age. The breast density was calculated to be 14%, 9%, and 6%, respectively. The decrease in volumetric breast density follows an exponential trend, based on the data shown in Figure 3. The images are spatially registered to each other, meaning the breast positioning relative to the x‐ray tube and detector is identical across time points.

**FIGURE 11:**
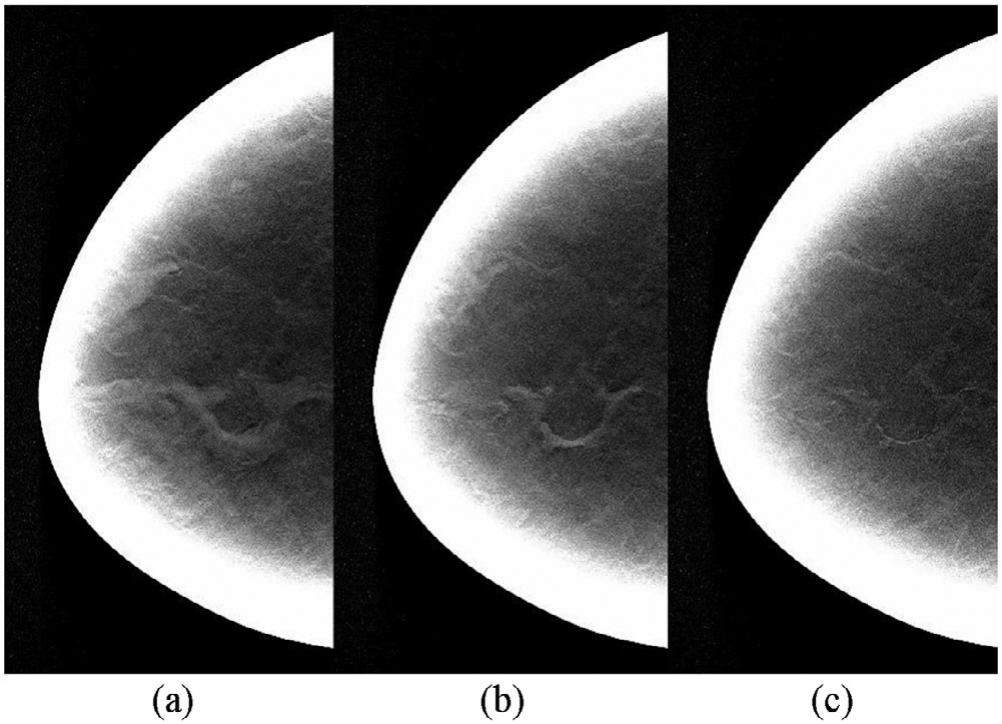
The reconstructed DBT slices of the phantoms from Figure 10, corresponding to different ages of a virtual woman at (a) 40, (b) 57, and (c) 74 years of age.

Shown in Figure 10 are the central slices in the voxel‐based phantom of a virtual woman at the ages of 40, 57, and 74 years. For this case example, we applied Method A (i.e. using the average annual change in VBD% for the entire population and assuming an exponential decline, as seen in Figure 3) to estimate the density at the given ages. Figure 11 shows the corresponding central reconstructed DBT image. The phantoms were not repositioned between scans, to isolate and visualize changes in tissue structures.

**FIGURE 12:**
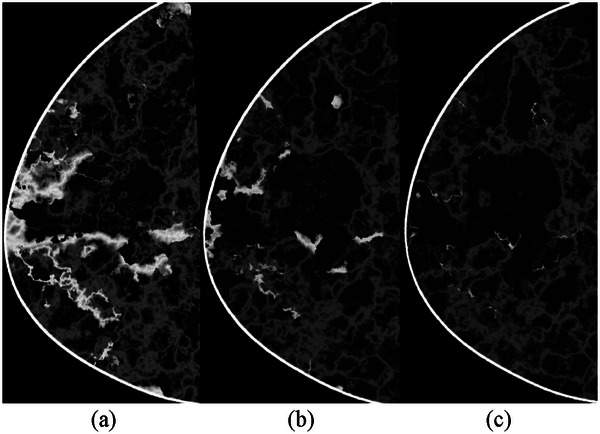
Cross‐sections of the same voxel‐based breast phantoms as shown in Figures 10 and 11, representing the same virtual woman at ages 40, 57 and 74. The phantoms have been repositioned relative to the imaging system by applying random angular rotations in all three planes (*x*, *y*, *z*). The rotation angles relative to the default position, seen in Figure 10, are: (a) 3°, 3°, 3°; (b) 8°, 5°, 1°; and (c) 10°, 10°, 3°.

Figures 10 and 11 show images of the breast phantoms consistently aligned relative to the imaging system. To demonstrate that the framework supports repositioning of the breast phantoms relative to the detector and imaging geometry, we present the same cross‐section of the same phantoms at different orientations, mimicking variations in breast positioning between imaging sessions (Figure 12).

### 4.5 Breast tumor changes over time

**FIGURE 13:**
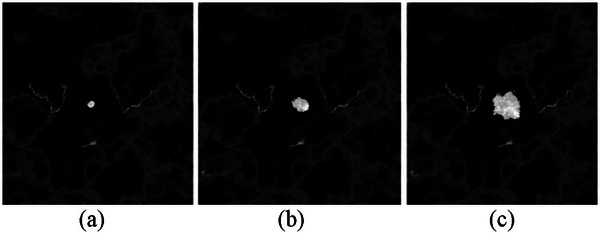
Central slices of the voxel‐based phantom, illustrating a simulated tumor at (a) 5 mm at the initial time point, (b) 10 mm 282 days later, and (c) 18 mm 2 years later. We assumed a tumor volume doubling time of 282 days.

**FIGURE 14:**
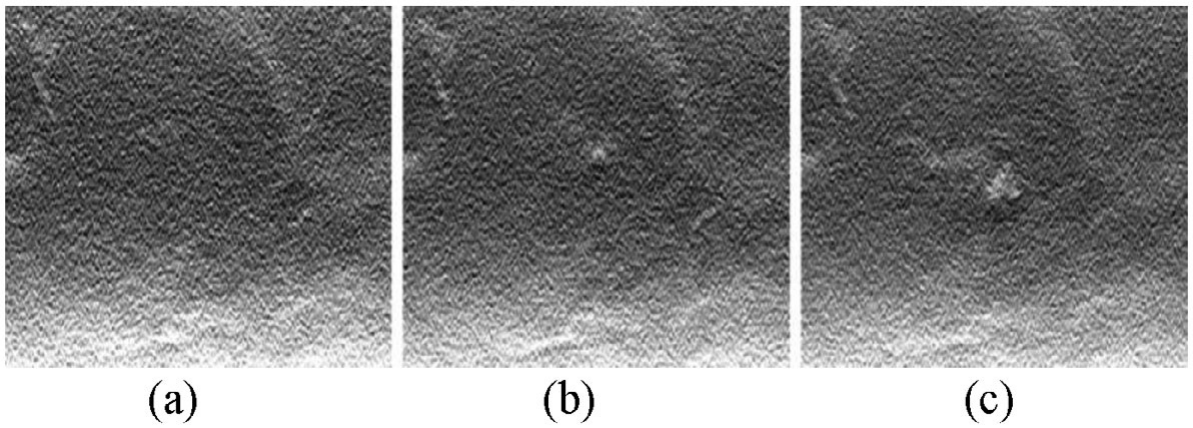
The corresponding reconstructed DBT slice of the simulated tumor shown in Figure 13.

Figure 13 shows a cropped central slice of the voxel‐based phantom, illustrating a small lesion at three different time points, as shown in Figure 5. Figure 14 shows the corresponding central slice in the reconstructed DBT image. At the initial time point (a), the tumor is 5 mm in diameter, barely visible in the reconstructed image. At time point (b), 282 days after the initial time point, the tumor has doubled in size (assuming a doubling time of 282 days). Time point (c) shows the tumor 2 years after the initial time point, having grown to 18 mm in diameter, that is, at the time of the second screening round.

## 5 DISCUSSION

We present STELLA‐R, a simulation framework based on the Perlin noise algorithm to simulate background breast anatomy and soft tissue breast lesions. We introduce two applications of our simulation framework, aimed at simulating changes in breast density and the growth of soft tissue breast lesions in voxel‐based phantoms. Our work is an additional step toward reducing the need for large‐scale clinical imaging trials, as it enables longitudinal virtual studies. This encourages the use of virtual studies to further explore, for example, disease progression and treatment effects over time. Moreover, our simulation framework enables customization of breast phantoms based on real‐world data. This opens possibilities for tailored simulation cohorts. This customization is, however, dependent on the availability of existing data sets. Our current framework has been customized using the Sweden‐based screening reference population from the MBTST (referred to in this work as the real‐world DM screening population).^12^, ^13^ The framework is adaptable for representing diverse cohorts globally by fine‐tuning it with relevant clinical data.

We have shown that we are able to model the dependence between several key variables such as breast volume, dense volume, age, change in breast volume, and change in breast density. The quantitative results (Tables 4 and 5) allow for a direct comparison of the multivariate structure in the real and virtual datasets, including comparisons of both marginal distributions and pairwise dependencies. We were able to show that means and standard deviations of each variable were preserved in the virtual dataset compared to the real‐world data. The pairwise dependence structure was also well captured, as most real‐world correlations (*τ*_r_) fell within the 95% confidence intervals of the simulated correlations (*τ*_s_). Overall, the real‐world and virtual data plots (Figure 6) show good visual correspondence, suggesting that the simulation captures the general patterns of the real‐world dataset well. However, a notable visual discrepancy arises in the age histograms: the real‐world data shows a pronounced spike at age 40, which is not reflected in the virtual data. This spike may have several explanations. One possibility could be that many women begin the screening program at age 40, but discontinue participation over time or attend irregularly. As a result, the total number of participants naturally stabilizes with increasing age. Additionally, during the data collection period, the Swedish National Board of Health and Welfare lowered the recommended starting age for screening from 50 to 40. The way the invitations were conducted as part of this change may also be reflected in the data, likely contributing to the higher number of 40‐year‐olds. As a result, the distribution is skewed, and this spike alters the overall shape of the age distribution in the real‐world data. Several variable pairs, particularly breast volume and dense volume (Figures 6 and 7) exhibit tail dependence, which the t‐copula can effectively capture. While tail dependence is not strictly necessary to use a t‐copula, these observations support its use for modeling the overall dependence structure.

In our framework, age is included as part of the multivariate t‐copula distribution, capturing realistic correlations between age, breast density, and breast volume. Conditional sampling can be used to simulate specific ages, though the current model is fitted to a clinical screening population aged 40–74, so sampling should remain within this range for realistic results. The framework is flexible and can be adapted to other datasets to simulate different age ranges or population characteristics.

While the change in breast density and breast volume sampled from the copulas are suitable for simulating a single follow‐up time point, it becomes less reliable for longitudinal simulation studies, as the variability in the copula‐based changes can introduce excessive noise since they are based on two (close in time) time point measurements. As a result, when simulating breast development over multiple years, we are currently limited to using average changes in breast volume and density, either across the entire population (Figures 3 and 4) or stratified by age groups (Tables 1 and 2).

In the future, we aim to develop regression models capable of predicting breast volume and density continuously across any age. However, building such models requires richer longitudinal data with multiple follow‐up time points, beyond the two currently available. The Malmö Breast ImaginG (M‐BIG) database,^39^ could offer such longitudinal data by tracking real women over extended periods. M‐BIG includes previously collected DM and DBT examinations performed in Malmö, Sweden from 2004 to 2020. This long‐term follow‐up is not only essential for developing accurate regression models but also critical for improving the realism and reliability of our simulations. We therefore emphasize that this study represents an initial framework for simulating longitudinal changes in breast density. The current results primarily demonstrate the feasibility of simulating these changes, but further work and additional longitudinal data is required to assess the accuracy of simulations at each time point.

The overall volumetric breast densities in our simulated cases (7%–52%) seem slightly excessive compared to the clinically reported range of volumetric density (∼2%–35%).^40^, ^41^ The absolute percentage of dense tissue in our phantoms is determined by weight factors assigned to the materials representing the simulated tissue structures. Fine‐tuning of the material weights is ongoing. In reality, breast density is also likely to be unevenly distributed across quadrants,^42^ and the risk of tumor development is primarily influenced by the local density.^43^ However, since our method does not fully account for the spatial distribution of density within the breast, tumors were placed based on quadrant‐specific onset probabilities and a linear relationship between tumor risk and local density.

Due to limitations in controlling the spatial distribution of dense tissue, the simulations do not currently replicate BI‐RADS density categories. Ongoing work aims to improve control over both the spatial distribution and Perlin noise parameters to better manage simulated densities. In the meantime, we recommend interpolating between the presented threshold levels/scaling factors (Figure 8) to estimate intermediate volumetric breast densities.

We chose an exponential relationship to describe the evolution of breast tissue over time, in line with previous studies that reported exponential or stepwise patterns.^32^, ^34^, ^35^ Commonly, the volumetric breast density is measured globally, as an average over the whole breast. Local changes in the density have not been extensively investigated. It would be of interest to try to simulate not only the volumetric breast density but also the distribution and texture from real cases. In contrast, a linear relationship was used to model tumor progression over time. As outlined in previous research, tumor growth follows a linear pattern during a clinical screening window.^14^ For larger tumors, growth may slow down as the tumor reaches its size limitations, influenced by factors such as reduced oxygen and nutrient supply. On the other hand, very small tumors at the cellular level may exhibit exponential growth. However, for tumors within the typical screening size range (5–20 mm in diameter), the growth rate has been observed to follow a largely linear pattern.^14^ In our simulation model, the tumor growth is independent of the woman's age. While age could theoretically influence growth rate, for example, older women may develop slower growing tumors, this effect is not included in the present simulations.

As for any modeling, improving realism and computational efficiency is a permanent ongoing task. We have previously compared clinical real‐world ROIs of breast parenchyma with simulated ROIs generated using Perlin noise, demonstrating that between 22% and 72% of simulated images were mistaken for real, and 23%–45% of real images were rated as simulated.^44^ We have also shown that our Perlin noise algorithm can generate lesions with clinically relevant shape and margin characteristics, with a plausible realism.^6^ This supports the use of Perlin noise for generating realistic simulations of breast anatomy; however, more extensive human observer studies are currently underway to further validate these findings. Specifically, validation is needed to fully capture the structural evolution of tumors in longitudinal imaging. Currently, because lesions are embedded within the background tissue, their interiors appear relatively stable across simulated time points. The added Perlin noise introduces variability that can mimic structural changes, such as evolving vasculature. By simulating the lesions with the same parameters, lesion margins can be largely preserved when diameter is increased, with minor natural shape variation. However, further studies are needed to determine how closely these changes reflect true tumor evolution. The current pipeline does not yet include simulations of microcalcifications, architectural distortions, or other cancer types, though this is an area of ongoing investigation. Nonetheless, there are toolboxes available for simulating for example, microcalcifications that could be integrated into our framework.^45^

To model structural changes in the breast, tissue involution was simulated by applying thresholding to the Perlin noise. This procedure removes certain glandular structures entirely while leaving others unchanged, thereby mimicking the selective disappearance of dense regions over time. Although this represents a simplified mechanism, it can be interpreted as an approximation of tissue involution, where glandular elements regress and are progressively replaced by fat tissue. While it does not reproduce the full histological complexity of tissue involution, the approach captures its imaging manifestation, namely the regional loss of glandular tissue visible on mammography.

Ongoing work includes exploring methods to reduce simulation time, such as using pre‐generated Perlin noise volumes instead of generating them on the fly, or adopting more computationally efficient noise functions.^46^ Our future work is also focused on a continuous refinement of the simulated tissue. The first approach is to enhance our Perlin noise model to incorporate directionality, meaning that the tissue components will visually progress in the same direction. This is commonly observed in real clinical cases, as the dense regions and the tissue directionality converge toward the nipple area. The model as of now includes no simulation of compression, but we are actively developing a finite element‐based compression module which uses local elastic properties (Markbo et al., under revision). We also aim at further integrating the tumors with the tissue background, for example, by using the same set of frequency components in the tumor as in the tissue. Lastly, additional observer studies are necessary to validate our results with real clinical cases.

## 6 CONCLUSIONS

We have presented STELLA‐R, a simulation framework for simulating temporal changes of breast phantoms based on real‐world data. Our work supports the use of virtual imaging trials over time.

## CONFLICT OF INTEREST STATEMENT

AT has a research grant from Siemens Healthineers. SZ has received speaker's fees and travel support from Siemens Healthineers.
